# Effect of the Symbiosis with *Mycoplasma hominis* and *Candidatus* Mycoplasma Girerdii on *Trichomonas vaginalis* Metronidazole Susceptibility

**DOI:** 10.3390/antibiotics11060812

**Published:** 2022-06-16

**Authors:** Valentina Margarita, Le Chi Cao, Nicholas P. Bailey, Thuy Ha Thi Ngoc, Thi Minh Chau Ngo, Phuong Anh Ton Nu, Nicia Diaz, Daniele Dessì, Robert P. Hirt, Pier Luigi Fiori, Paola Rappelli

**Affiliations:** 1 Department of Biomedical Sciences, University of Sassari, Viale San Pietro 43/B, 07100 Sassari, Italy; vmargarita@uniss.it (V.M.); ndiaz@uniss.it (N.D.); danieled@uniss.it (D.D.); fioripl@uniss.it (P.L.F.); 2 Department of Parasitology, Hue University of Medicine and Pharmacy, 06 Ngo Quyen Street, Hue 49000, Vietnam; lechicao@hueuni.edu.vn (L.C.C.); hathingocthuy@hueuni.edu.vn (T.H.T.N.); ntmchau@hueuni.edu.vn (T.M.C.N.); tnphuonganh@hueuni.edu.vn (P.A.T.N.); 3 Biosciences Institute, Faculty of Medical Sciences, Newcastle University, Newcastle upon Tyne NE2 4HH, UK; n.bailey2@newcastle.ac.uk (N.P.B.); robert.hirt@newcastle.ac.uk (R.P.H.); 4 Mediterranean Centre for Disease Control (MCDC), 07110 Sassari, Italy

**Keywords:** metronidazole, resistance, *Trichomonas vaginalis*, *Mycoplasma hominis*, *Candidatus* Mycoplasma girerdii, RNA-seq

## Abstract

Trichomoniasis, the most common non-viral sexually transmitted infection worldwide, is caused by the protozoon *Trichomonas vaginalis.* The 5- nitroimidazole drugs, of which metronidazole is the most prescribed, are the only effective drugs to treat trichomoniasis. Resistance against metronidazole is increasingly reported among *T. vaginalis* isolates. *T. vaginalis* can establish an endosymbiosis with two *Mycoplasma* species, *Mycoplasma hominis* and *Candidatus* Mycoplasma girerdii, whose presence has been demonstrated to influence several aspects of the protozoan pathobiology. The role of *M. hominis* in *T. vaginalis* resistance to metronidazole is controversial, while the influence of *Ca.* M. girerdii has never been investigated. In this work, we investigate the possible correlation between the presence of *Ca.* M. girerdii and/or *M. hominis* and the in vitro drug susceptibility in a large group of *T. vaginalis* isolated in Italy and in Vietnam. We also evaluated, via RNA-seq analysis, the expression of protozoan genes involved in metronidazole resistance in a set of syngenic *T. vaginalis* strains, differing only for the presence/absence of the two *Mycoplasmas*. Our results show that the presence of *M. hominis* significantly increases the sensitivity to metronidazole in *T. vaginalis* and affects gene expression. On the contrary, the symbiosis with *Candidatus* Mycoplasma girerdii seems to have no effect on metronidazole resistance in *T. vaginalis*.

## 1. Introduction

Trichomoniasis is the most common non-viral human sexually transmitted disease in the world with 156 million new cases per year [1]. It is caused by *Trichomonas vaginalis*, a flagellated protist lacking a cyst stage. In women, the infection is often asymptomatic and the usual symptoms include vaginal discharge, spotting, and bleeding [2], while in men it usually presents as a transient asymptomatic infection, but can be associated with urethritis and prostatitis [3]. The presence of *T. vaginalis* in the urogenital tract has been also associated with the persistence of most carcinogenic HPV types, cervical and prostate cancer, and a higher risk of HIV and HPV infection [4,5].

The treatment of trichomoniasis was introduced only in 1959 through the use of metronidazole (MTZ), a 5-nitroimidazole derivative that, together with tinidazole, represents the only drugs known so far to be effective against infection [6]. Nitroimidazole resistance was reported for the first time in 1962 [7], and since then an increment of rates of resistance among *Trichomonas vaginalis* isolates has been reported [8].

Metronidazole enters the trichomonad cell in an inactive form through passive diffusion, reaching the hydrogenosomes [9]. Activation occurs through the reduction of MTZ in its toxic nitro-radical form and through competition with the enzyme hydrogenase as an electron acceptor from ferredoxin. In the presence of oxygen, toxic radicals are converted back to the inactive prodrug. The aerobic resistance observed in clinical *T. vaginalis* isolates can be caused by a reduced oxygen scavenging capacity that leads to higher oxygen concentrations inside the hydrogenosomes, thus decreasing drug effects. A reduced expression of the enzyme flavin reductase (FR1), which is part of the protozoan oxygen defense system, has been observed in metronidazole resistant *T. vaginalis* [10]. Resistance has also been associated with a downregulation of hydrogenosomal genes coding for metronidazole-activating enzymes such as pyruvate:ferredoxin oxidoreductase (PFOR) [11]. 

The percentage of *T. vaginalis* isolates resistant to MTZ varies from 2 to almost 10% depending on their geographic origin [11,12,13,14]. In fact, the true proportion is unknown since the susceptibility to metronidazole of *T. vaginalis* isolated strains is performed only in specialized laboratories mainly for research purposes.

An intriguing feature in *T. vaginalis* biology is its ability to establish stable relationships with other microorganisms of the vaginal microenvironment. In fact, in 1998 a symbiotic relationship between *T. vaginalis* and the bacterium *Mycoplasma hominis* was demonstrated. The relationship is highly species-specific. In fact, neither *Mycoplasma genitalium* nor *Ureaplasma spp.* have been detected to be in association with *T. vaginalis* [15]. Since then, several groups have investigated the association rate between the two microorganisms, showing that the percentage of *T.vaginalis* hosting *M. hominis* greatly varies depending on the geographical setting of the studies [16]. *M. hominis* is an obligate parasite of the human urogenital tract belonging to the class *Mollicutes*, the smallest organisms capable of independent replication. The infection is, in most cases, asymptomatic, and it can be associated with alterations of the vaginal microbiota and bacterial vaginosis [17]. 

The biological association between *T. vaginalis* and *M. hominis* has been shown to have a relevant impact on several aspects of the pathogenesis of both microorganisms. In fact, *M. hominis* receives protection from antibiotics and host immune response through intracellular localization in trichomonad cells, while *T. vaginalis,* in turn, increases its cytopathic activity, host cell damage, phagocytosis, and proinflammatory response [18,19]. 

*M. hominis* can colonize uterus and placental membranes in pregnant women, causing severe complications, such as preterm birth and chorioamnionitis [20]. The same adverse pregnancy outcomes have also been correlated with the presence of *T. vaginalis,* that, differing from *M. hominis*, is unable to reach the amniotic fluid [21,22]. Interestingly, *T. vaginalis* may function as a protected niche and act as a “Trojan horse” for *M. hominis.* Metronidazole treatment during pregnancy could induce a massive release of *M. hominis* from trichomonad cells that can reach placental membranes and amniotic fluid, leading to severe pregnancy sequelae [23]. 

Through metagenomic analyses a novel *Mycoplasma* species, initially named “Mnola” and later renamed *Candidatus* Mycoplasma girerdii, has been described in association with *T. vaginalis* [24]. Interestingly, the DNA of *Ca.* M. girerdii was found almost exclusively in the vaginal discharge of *T. vaginalis*-infected women [25]. The relevance of this new unculturable *Mycoplasma* species regarding the pathobiology of *T. vaginalis* is still largely unknown. 

The possible role of the symbionts in the development of metronidazole resistance of *T. vaginalis* has been, so far, poorly investigated. In fact, the effect of *M. hominis* presence in association with *T. vaginalis* is controversial: while some studies suggest a positive association between the presence of bacteria in *T. vaginalis* and resistance to metronidazole [26,27], other works report a lack of correlation between *M. hominis* presence and drug resistance [28,29,30]. Moreover, the consequences of the presence of the new mycoplasma species *Ca.* M. girerdii on metronidazole sensibility in *T. vaginalis* strains have never been studied. Thus, for the first time, we investigated the prevalence of *Ca.* M. girerdii and *M. hominis* in *T. vaginalis* strains isolated in two distant geographical areas—Italy and Vietnam—and tested their sensitivity to MTZ to shed light on the possible correlation between the presence of one or both species of *Mycoplasma* and the in vitro drug susceptibility. Then, to overcome any influence of trichomonad strain-to-strain variability, we produced a set of syngenic *T. vaginalis* differing only for the presence/absence of one or both *Mycoplasma*, and we evaluated the expression of protozoan genes involved in MTZ resistance via RNA-seq analysis. 

## 2. Results

### 2.1. Mycoplasma Hominis and Candidatus Mycoplasma Girerdii Detection in T. vaginalis Isolates

In this study, 47 strains of *T. vaginalis* isolated in Vietnam and 17 *T. vaginalis* strains isolated in Italy were screened by PCR for the presence of *M. hominis* (Mh) and *Ca.* M. girerdii (Mg). We found *M. hominis* in 32% of *T. vaginalis* strains isolated in Vietnam and in 76.5% of strains from Italy, while *Ca.* M. girerdii was present in 19% of the Vietnamese and 59% of the Italian isolates. Interestingly, a high rate of contemporary presence of the two *Mycoplasma* species was observed, especially in Italian strains (47%). The 62% of Vietnamese strains were totally *Mycoplasma*-free, while, among Italian strains, the percentage decreased to 12%. (Table 1).

### 2.2. Metronidazole Susceptibility of T. vaginalis Isolates

All 64 *T. vaginalis* isolated were tested for their in vitro susceptibility to MTZ. The minimum lethal concentration (MLC) was assessed in aerobic conditions after 48 h of incubation at 37 °C. The overall MLC mean value was 4.6 µg/mL; higher in Vietnamese strains (mean 5.0 µg/mL ± 4.5) than in the Italian ones (mean 3.2 µg/mL ± 4.18). Five strains (8%) showed a reduced susceptibility to metronidazole, with an MLC value of 17.1 µg/mL. Interestingly, 4 out of 5 *T. vaginalis* strains showing lower sensitivity to metronidazole were isolated from Vietnam (Table 2).

### 2.3. Association between Symbionts Presence and Metronidazole Sensitivity in T. vaginalis 

**Figure 1:**
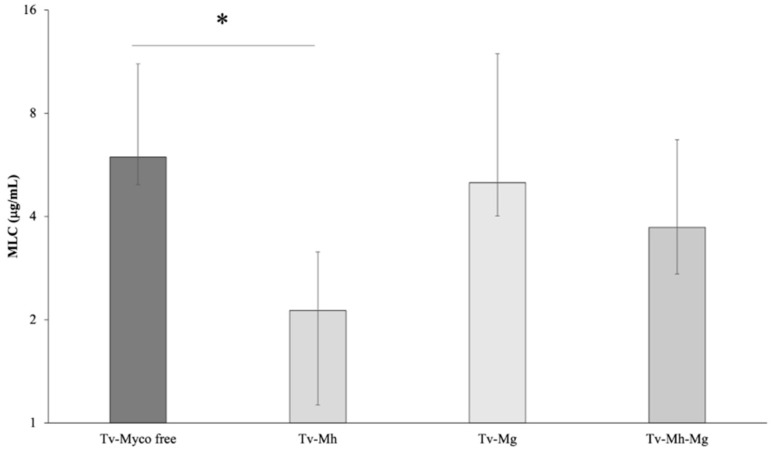
The impact of *M*. *hominis* and ‘*Ca*. M. girerdii’ presence on sensibility of metronidazole of *T. vaginalis* isolates. Statistical significance was tested via Student’s *t*-test. Tv *= T. vaginalis*, Mh = *M. hominis*, Mg = *Ca.* M. girerdii. *M. hominis* positive strains are associated with an increase of sensitivity to metronidazole compared to *M.hominis* free strains, independently from the presence of Mg (* *p* < 0.01).

The association between the presence of *M. hominis* and/or *Ca.* M. girerdii and the sensitivity to metronidazole in the 64 *T. vaginalis* isolates was evaluated (Figure 1). A statistically significant difference in the mean MLC values of *M. hominis-*infected *T. vaginalis* compared with non-infected strains was observed (*p* < 0.01), with the MLC mean value of Mh-free isolates two-fold higher than the MLC average of *T. vaginalis* strains harbouring *M. hominis*. On the contrary, the presence of *Ca.* M. girerdii seemed to not influence the sensitivity to MTZ of *T. vaginalis* strains (*p* = 0.55). 

These results suggest a possible correlation between *M. hominis* symbiosis and metronidazole susceptibility in *T. vaginalis* strains. Interestingly, 4 out of 5 *T. vaginalis* strains showing a reduced sensitivity to metronidazole (MLC value ≥ 17 µg/mL) are totally *Mycoplasma* free.

### 2.4. Sensitivity of Isogenic T. vaginalis Strains to Metronidazole in Aerobic Conditions

**Figure 2:**
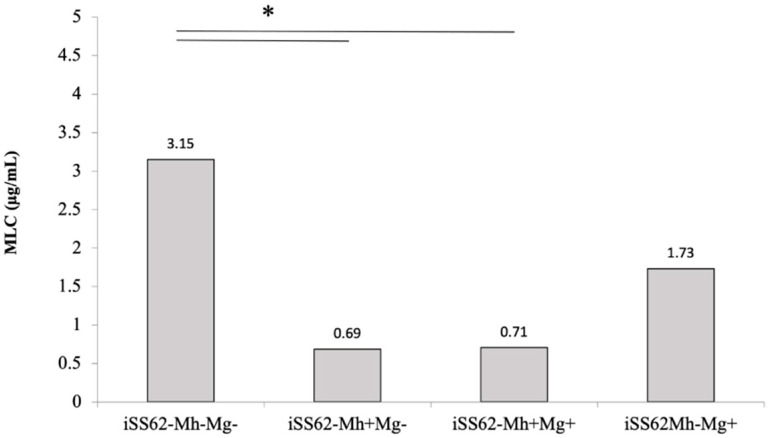
The metronidazole susceptibility of *T. vaginalis* isogenic strains. The mean values of metronidazole MLC for *T. vaginalis* experimentally (iSS62-Mh^+^Mg^−^, iSS62-Mh^+^Mg^+^) and naturally *Mycoplasma*-infected (iSS62-Mh^−^Mg^+^), and for *Mycoplasma*-free *T. vaginalis* (iSS62-Mh^−^Mg^−^), were compared. The presence of *M. hominis* in trichomonad cells is associated with an increase of sensitivity to metronidazole compared to iTv, independently from the presence of Mg (* *p* < 0.05). Statistical significance was tested by Student’s *t*-test.

In order to eliminate any strain-to-strain variability among trichomonad isolates, we generated a set of isogenic *T. vaginalis* strains differing only in the presence/absence of one or both Mycoplasma species, as assessed by PCR: iSS62-Mh^+^Mg^+^, iSS62-Mh^+^Mg^−^, iSS62-Mh^−^Mg^+^and iSS62-Mh^−^Mg^−^. A metronidazole sensitivity assay was then performed on the four *T. vaginalis* isogenic strains. As shown in Figure 2, the presence of *M. hominis* in both iSS62-Mh^+^Mg^+^, iSS62-Mh^+^Mg^−^ caused a 4.5-fold reduction of MLC values compared with iSS62-Mh^−^Mg^−^ (*p* < 0.05). On the contrary, although the iSS62-Mh^−^Mg^+^ strain showed a slightly lower sensitivity to the drug compared with iSS62-Mh^−^Mg^−^ (*p* = 0.1061), the presence of *Ca.* M. girerdii in *T. vaginalis* does not significantly influence the sensitivity to MTZ.

### 2.5. Expression of Genes Associated with Drug Resistance in T. vaginalis Isogenic Strains

**Figure 3:**
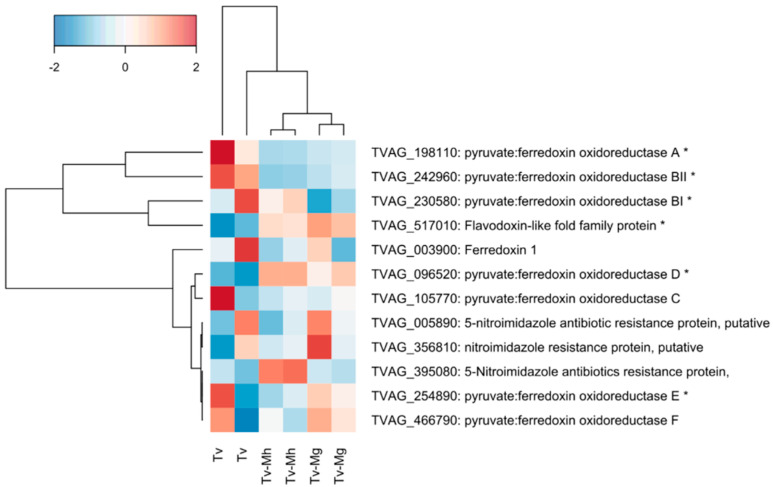
The differential expression of selected *T. vaginalis* genes related to metronidazole resistance alone and in association with *M. hominis* or with *Ca.* M. girerdii. Expression units are z-scaled trimmed mean of M-values (TMM). * Indicates genes with a statistically significant difference of expression between *Mycoplasma-*free *T. vaginalis* iSS62-Mh^−^Mg^−^ (Tv) and *T. vaginalis* associated with *M. hominis* iSS62-Mh^+^Mg^−^ (Tv-Mh).

Through gene ontology (GO) enrichment analysis, we investigated selected genes associated with metronidazole resistance in *T. vaginalis* (Supplementary Table S1) and observed a slightly significant decrease in the expression of two pyruvate: ferredoxin oxidoreductase (PFOR) genes; namely, PFOR A (TVAG_198110) and PFOR BII (TVAG_242960), in *T. vaginalis* associated with *M. hominis* (iSS62-Mh^+^Mg^−^) compared with *Mycoplasma-*free *T. vaginalis* iSS62-Mh^−^Mg^−^. The average log_2_ fold change value for the two genes was, respectively, −1.61 (*p*-value 0.0002) and −1.39 (*p*-value 0.00001) (presence/absence of *M. hominis*). PFOR A and PFOR BII were also significantly downregulated in the presence of *Ca.* M. girerdii compared with the control (respective log_2_ fold changes were −1.26 and −1.02, with p-values of 0.001 and 0.0002). Intriguingly, *T. vaginalis* PFOR D (TVAG_096520) showed the opposite regulatory profile and was significantly upregulated during co-culture with *Ca.* M. girerdii compared with the control (log_2_ fold change 2.29; *p*-value 0.00001). The expression of flavin reductase 1 (TVAG_517010) was also upregulated in iSS62- Mh^+^Mg^−^ (log_2_ fold 0.86, *p*-value 0.0007) and iSS62- Mh^−^Mg^+^ (log_2_ fold 1.02, *p*-value 0.0001). Finally, we did not detect any large differences in the expression of ferredoxin 1 (TVAG_003900, log_2_ fold −0.4, *p*-value 0.16) when *T. vaginalis* was infected by *M. hominis*, as previously reported [27], (Figure 3). 

## 3. Discussion

Metronidazole and tinidazole are currently the only drugs officially used for the treatment of trichomoniasis, the most common non-viral sexually transmitted infection worldwide. A recent review reported that the global prevalence of *T. vaginalis* is resistant to MTZ ranges between 2.2−9.6% [11]. In recent years, several research groups investigated *T. vaginalis* MTZ resistance, but the underlying molecular mechanisms are not yet fully understood. Metronidazole enters the trichomonad cell in an inactive form through passive diffusion and needs to be reduced to its active nitro radical anion form in order to damage *T. vaginalis*. MTZ enters the hydrogenosome and competes with hydrogenase for electrons from ferredoxin, turning into the reduced active drug. Under aerobic conditions, oxygen converts MTZ back into its inactive form. Impaired oxygen scavenging and energy-production pathways have been linked to aerobic metronidazole resistance [9,30].

Interestingly, *T. vaginalis* is able to establish a stable symbiosis with two Mycoplasma species. The presence of the bacterium *Mycoplasma hominis,* an obligate human parasite colonizing the lower urogenital tract, has been demonstrated since 1998 in trichomonad isolates from different geographical areas, and, more recently, a new Mycoplasma species known as *Ca.* Mycoplasma girerdii, has been observed via metagenomic analysis almost exclusively in the vaginas of women infected by *T. vaginalis.* Several studies have demonstrated that the presence of the symbiont *M. hominis* within trichomonad cell has important consequences for the pathobiology of the protozoon [22], while the possible effect of the presence of *Ca.* Mycoplasma girerdii on trichomonad features has only recently been studied [31]. The possibility that the symbiosis could interfere with the resistance to MTZ of *T. vaginalis* isolates has only been investigated for *M. hominis,* leading to contradictory results [27,28,29,30].

In the present work, we investigated the possible correlation between the presence of the two Mycoplasma symbionts and the in vitro drug susceptibility in a large group of *T.vaginalis* isolated in Italy and Vietnam. 

In our study 31.9% and 76.5% of *T. vaginalis* strains, respectively, isolated in Vietnam and Italy, were infected by *M. hominis*, confirming the high infection rate variability in different geographic areas. In fact, since the first observations of Rappelli and colleagues [15], several groups used PCR to demonstrate the presence of *M*. *hominis* in the trichomonad isolates of different geographic origins, with infection rates ranging from a minimum of 5% observed in Cuba [32] to over 89% detected in Italy [15]. We have also verified the presence of *Ca.* M. girerdii in our protozoan isolates, with and without *M. hominis*. Until now, the new uncultured *Mycoplasma* species had been mostly observed in the vaginal secretions of women affected by trichomoniasis through 16S rRNA microbial surveys and metagenomic analyses, but not in pure *T. vaginalis* cultures [24,25]. In fact, Ioannidis et al. [33] observed *Ca.* M. girerdii in 32 out of 100 cultured *T. vaginalis* strains from Greece, but the contemporary presence of both symbionts in a single *T. vaginalis* isolate is not described. We found that 19.8% of Vietnamese and 58.8% of Italian trichomonad strains were positive for *Ca.* M. girerdii. Interestingly, 13% of Vietnamese strains and 47% of Italian strains are infected by both *Mycoplasma*. Fettweis et al. observed, via metagenomic analysis, the contemporary presence of *Ca.* M. girerdii and *M. hominis* in 67% of the vaginal samples from African American women affected by acute trichomoniasis [25]. Our results confirmed for the first time the contemporary presence of both Mycoplasma species in a single trichomonad isolate. The percentage of *T. vaginalis,* totally free of *Mycoplasma,* greatly differs in the two groups, being 62% among the Vietnamese strains and only 12% among the Italian ones.

To establish if the presence of the two symbionts could influence the sensibility to metronidazole, *T. vaginalis* isolates were tested for in vitro metronidazole resistance in aerobic conditions. All 64 strains tested had a result of sensitivity to metronidazole, with 75% of isolates showing MLC values ≤ 4.3 µg/mL. Altogether, Vietnamese strains show a higher mean MLC value compared to the Italian ones (5.0 µg/mL versus 3.2 µg/mL). Moreover, among the five *T. vaginalis* strains showing a reduced sensitivity (MLC value 17.1 µg/mL), four were isolated in Vietnam. To our knowledge, this is the first report on the metronidazole susceptibility of *T. vaginalis* isolated in Vietnam. 

In our study, the presence of the symbiont *M. hominis* is positively related to a higher sensitivity to MTZ in *T. vaginalis*: the mean MLC was 2.9 µg/mL in Mh-positive and 5.9 µg/mL in Mh-negative strains (*p* < 0.05). Moreover, we observed that 4 out of 5 isolates showing a reduced sensitivity to MTZ are *Mycoplasma* free. Our results contrast with those obtained by Xiao et al. in 2006, who reported an increased resistance in protozoa infected by *M. hominis* [26]. More recently, other groups reported the absence of an association between metronidazole susceptibility and the presence of *M. hominis* in *T. vaginalis*. Butler et al. tested 55 isolates from the USA, reporting a slight, insignificant increase of resistance in the group of Mh negative *T. vaginalis* [28], and da Luz Becker et al. analyzed 30 trichomonad isolated in Brazil, obtaining similar results [30]. Interestingly, considering only MTZ resistant strains, 20 out of 24 isolates from the USA and 3 out of 4 from Brazil were *M. hominis* free. Notably, in our study 4 out of 5 strains with reduced sensitivity to MTZ were *Mycoplasma* free. Moreover, in a previous study we observed that the only *T. vaginalis* strain resistant to metronidazole (MLC > 50 µg/mL) was *M. hominis* free [34].

We investigated, for the first time, a possible relationship between infection by *Ca.* M. girerdii and trichomonad sensitivity to MTZ. Unlike *M. hominis*, the presence of *Ca.* M. girerdii seems to not influence drug resistance: MLC mean values were the same in *T. vaginalis* positive and negative for *Ca.* M. girerdii (4.6 µg/mL). 

In order to reduce the strain-to-strain variability characterizing *T. vaginalis* isolates, we artificially infected a naturally Mycoplasma-free *T. vaginalis* with one or both *Mycoplasma* species, obtaining four isogenic strains that were subjected to a metronidazole susceptibility assay. Results confirm data obtained with *T. vaginalis* clinical isolates: *Mycoplasma*-free *T. vaginalis* are more tolerant to MTZ than *T. vaginalis* associated with *M. hominis* or with both *Mycoplasmas* (*p* < 0.05). On the contrary, as observed with our clinical isolates, the presence of *Ca.* M. girerdii does not influence MTZ resistance, although strains artificially infected with *Ca.* M. girerdii showed a slightly reduced sensitivity to the drug compared with negative *T. vaginalis* (*p* = 0.1061). These findings suggest a correlation between *M. hominis* symbiosis and metronidazole susceptibility in *T. vaginalis* strains, supporting previous data where the presence of bacteria was associated with a lower MIC value of trichomonad cells compared with *Mycoplasma*-free isolates.

The metronidazole resistance in *T. vaginalis* strains has been associated to a decrease of the expression of hydrogenosomal proteins, including flavin reductase 1 (FR1), ferredoxin (Fdx), and pyruvate ferredoxin oxidoreductase (PFOR) [35]. 

In order to investigate if the presence of *Mycoplasma* species in trichomonad cells can modulate the expression of genes involved in MTZ resistance, we analyzed their mRNA levels in our isogenic strains by RNAseq analysis.

We observed a significant increase of flavin reductase1 expression in *T. vaginalis* infected with *M. homins* and with *Ca.* M. girerdii compared to the mycoplasma-free isogenic strain. In *T. vaginalis,* the enzyme plays a pivotal role in oxygen scavenging mechanisms, lowering intracellular oxygen concentrations and, consequently, reducing drug inactivation [10]. The increase of Fd1 expression can therefore explain, at least in part, the higher sensitivity to metronidazole observed in *T. vaginalis* infected by *M. hominis* in aerobic conditions. 

A decrease in the expression of two out of seven pyruvate:ferredoxin oxidoreductase (PFOR) genes, namely PFOR A and PFOR BII, was observed in *M. hominis* and in *Ca.* M. girerdii positive *T. vaginalis* compared with *Mycoplasma-*free *T. vaginalis*. In our experimental model, the presence of *M. hominis* is associated with an increased susceptibility to MTZ, suggesting that a down-regulation of PFOR is not necessarily associated with drug resistance. Our results are in agreement with previous studies, showing the absence of association between the down-regulation of PFOR and MTZ resistance in *T. vaginalis* [27,30], supporting the hypothesis that PFOR RNA expression is not necessarily linked to a resistance to metronidazole. The slight down-regulation of PFOR could simply be due to the presence of Mycoplasmas, as suggested by Fürnkranz and colleagues [27]. 

## 4. Materials and Methods

### 4.1. Culture Conditions of Trichomonas vaginalis Isolates 

A total of 64 *T. vaginalis* clinical isolates from women affected by acute trichomoniasis were analysed. Forty-seven isolates were collected by the Department of Parasitology of Hue University of Medicine and Pharmacy University and Reproductive Health Centre (Vietnam), and 17 isolates by the laboratory of Microbiology of University of Sassari (Italy). Trichomonad isolates were cultured in Diamond’s TYM medium supplemented with 10% FBS at 37 °C in a 5% CO_2_ atmosphere [36] for at least 15 days, and then preserved via freezing at −80 °C with FBS 10%, and adding 5% dimethylsulfoxide (DMSO) until use. Protozoa in an exponential growth phase, exhibiting a viability of >95%, were used in all experiments. 

### 4.2. Screening for Mycoplasma Species in T. vaginalis Isolates

Genomic DNA was extracted from all 64 *T. vaginalis* clinical isolates with DNeasy Blood & Tissue Kit (Qiagen Ltd., West Sussex, UK) according to the manufacturer’s protocols. The presence of *Mycoplasma* species in association with each *T. vaginalis* strain was assessed by PCR using the following 16S rRNA specific primers: for *M. hominis* has been used RNA H1 (5-CAATGGCTAATGCCGGATACGC-3) and RNA H2 (5-GGTACCGTCAGTCTGCAAT-3) primers [37], while for *Ca.* M. girerdii Forward has been used OTU_M1 forward (5-CATTTCCTCTTAGTGCCGTTCG-3) and OTU_M1 reverse CGGAGGTAGCAATACCTTAGC-3) primers [25]. The PCR cycle conditions for both mycoplasma gene amplification were 30 cycles each at 95 °C for 30 s, 62 °C for 1 min (for *M. hominis*) or 58 °C for 1 min (for *Ca.* M. girerdii), 72 °C for 30 s and a final extension at 72 °C for 10 min. PCR products were analysed in 10 or 1 min, and 72 °C for 1 min. PCR products were analysed by 1% agarose gel and viewed under a UV transilluminator: the presence of specific bands of 344 bp and 310 bp confirmed the presence of M. *hominis* and *Ca.* M. girerdii, respectively. 

### 4.3. Generation of Isogenic T. vaginalis Strains

Using isolate *T. vaginalis* SS62, we generated a set of isogenic strains differing only for the presence/absence of the two Mycoplasma species: iSS62-Mh^+^Mg^+^, iSS62-Mh^+^Mg^−^, iSS62-Mh^−^Mg^+^and iSS62-Mh^−^Mg^−^. 

Being SS62 naturally *Ca.* M. girerdii-infected (named thereafter as iSS62-Mh^−^Mg^+^), to generate iSS62-Mh^−^Mg^−^ we eliminated the bacteria by cultivating trichomonad cells for 7 days in a medium supplemented with Plasmocin™ (Invivogen, San Diego, CA, USA) at a final concentration of 25 μg/mL [27]. Then, the cells were cultivated in complete Diamond’s TYM medium without Plasmocin™ for a further 15 days. At the end of the treatment, the absence of bacteria was confirmed by PCR. 

To obtain isogenic *T. vaginalis* iSS62-Mh^+^Mg^+^ and iSS62-Mh^+^Mg^−^, strains iSS62-Mh^−^Mg^+^ and iSS62-Mh^−^Mg^−^ were stably infected with *M. hominis* isolate MhMPM2 as previously described [38,39]. One ml of an overnight culture of *M. hominis* (strain MPM2), corresponding to approximately 10^9^ colour-changing units (CCU), was added to a 10-mL mid-log phase culture of the *M. hominis*-free iSS62-Mh^−^Mg^+^ and iSS62-Mh^−^Mg^−^ for 5 days in a TYM complete medium. Parasites were then cultivated for a further 10 days with 1:16 daily passages in TYM complete medium. At the end of the treatment, 100 µL of each supernatant was plated on a solid BEA medium to verify the presence of viable *M. hominis*. The presence of single or double infections in *T. vaginalis* isogenic strains was confirmed by PCR.

### 4.4. Metronidazole Susceptibility Assay in Aerobic Conditions 

The 64 *T. vaginalis* clinical isolates and the four isogenic *T. vaginalis* strains (iSS62-Mh^+^Mg^+^, iSS62-Mh^+^Mg^−^, iSS62-Mh^−^Mg^+^ and iSS62-Mh^−^Mg^−^), obtained as described above, were tested to assess their metronidazole susceptibility in aerobic conditions, according to the methods previously described by other authors [27,28,29]. Assays were performed in 96-well flat-bottomed microtiter plates and 1 × 10^4^ exponentially growing cells were seeded in each well with increasing levels of metronidazole ranging from 0.2 to 200 μg/mL. After 48 h of incubation in 5% CO_2_, the plates were microscopically observed and the minimum lethal concentration (MLC), defined as the lowest drug concentration at which no motile trichomonads were observed at the end of the incubation period, was detected.

All experiments were performed twice in triplicate for each isolated test. Untreated cultures of each trichomonad strain were used as controls. *T. vaginalis* with minimal lethal concentrations ≤25 μg/mL are considered MTZ-sensitive [28].

### 4.5. RNA Preparation

RNA was extracted from *T. vaginalis* isogenic strains. For each condition, two aliquots of 2 × 10^6^ exponentially growing cells have been harvested, washed and resuspended in 700 μL of RNAlater (ThermoFisher Scientific, Waltham, MA, USA), then stored at −80 °C until use.

Samples were then thawed on ice, diluted with 0.7 mL nuclease-free PBS, and pelleted by centrifugation at 6 k× *g* for 5 min at 4 °C. RNA was extracted from the resulting pellet using TRIzol (ThermoFisher Scientific, Waltham, MA, USA) according to the manufacturer’s instructions, with some modifications. Briefly, TRIzol and chloroform were used to lyse cells and solubilise cell components, then RNA was precipitated, washed, and resuspended in 30 µL nuclease-free water.

RNA concentration was assessed by Qubit RNA High Sensitivity kit (ThermoFisher Scientific, Waltham, MA, USA), according to the manufacturer’s instructions. UV absorbance at 230 nm, 260 nm, and 280 nm was measured using a nanodrop 2000 c spectrophotometer as an indicator of purity. RNA integrity and absence of genomic DNA contamination were confirmed using a TapeStation System (Agilent, Santa Clara, CA, USA) with the resulting gel images and electropherograms manually examined. 

### 4.6. RNA Sequencing and Data Analysis

Library preparation and Illumina sequencing was performed by Novogene UK. A standard protocol was used to prepare libraries and the depletion of prokaryotic and eukaryotic ribosomal RNA (rRNA) was obtained via Ribo-Zero kit (Illumina). Approximately 50 million paired end reads per sample were generated using an Illumina NovaSeq 6000 platform. The read length was 150 bp and the insert size was from 250 bp to 300 bp. The sequences with low quality reads (reads with greater than 10% N (undetermined base) or over 50% of bases at or below 5 Phred quality score) were deleted. All the reads can be accessed via the BioProject entry PRJNA674783 at the NCBI [31].

The *T. vaginalis* G3 genome (Accession ASM282v1) [40] was used as the parasite reference sequence, and genes with an expression level of at least 1 transcript per million (TPM) were considered to be expressed. 

The edgeR package [41] was used to test for differential gene expression using the negative binomial generalised linear model with a quasi-likelihood test, considering only genes with a log_2_ fold change of greater than 1.2 for testing.

The gene ontology (GO) enrichment analysis PANTHER [42] was used, a set of *T. vaginalis* genes potentially associated to metronidazole sensibility was used as a reference database, and uninformative and redundant enriched functions were removed manually. KEGG enrichment analysis was performed using edgeR [41]. For significance tests of differential gene expression and functionally enriched KEGG pathways and GO functions, *p*-values were adjusted using the false discovery rate/Benjamini–Hochberg (FDR/BH) method.

### 4.7. Statistical Analysis

A statistical analysis was performed using MS Excel for a chi-square test and for a Student’s *t*-test. A *p* < 0.05 was considered significant.

## 5. Conclusions

Our findings demonstrate the contemporary presence of both the symbionts *Ca.* Mycoplasma girerdii and *M. hominis* in *T. vaginalis* isolates and confirm the high differences in prevalence existing between different geographical areas. Moreover, our data strongly suggest that the presence of *M. hominis* is associated with a reduced resistance to metronidazole in *T. vaginalis* and affects gene expression. We also observed, for the first time, that, conversely, the symbiosis with *Candidatus* Mycoplasma girerdii seems to have no effect on metronidazole resistance in *T. vaginalis*. Further investigation of a larger group of isolates is needed to shed light on the factors underlying the capability of *M. hominis* to interfere with antimicrobial resistance in *T. vaginalis.*

## Figures and Tables

**Table 1:** The occurrence of *M. hominis* and *Ca.* M. girerdii among *T. vaginalis* strains isolated in Vietnam and Italy.

Microbial Association	*T. vaginalis* (Vietnam) N (%)	*T. vaginalis* (Italy) N (%)
*T.vaginalis* Mh^neg^ Mg^neg^	29 (62%)	2 (12%)
*T.vaginalis* Mh^pos^ Mg^neg^	9 (19%)	5 (29%)
*T.vaginalis* Mh^neg^ Mg^pos^	3 (6%)	2 (12%)
*T.vaginalis* Mh^pos^ Mg^pos^	6 (13%)	8 (47%)

**Table 2:** The MLC value of metronidazole among *T. vaginalis* strains isolated from Vietnam and Italy. In vitro antimicrobial susceptibility of 64 *T. vaginalis* isolated strains was evaluated, and minimal lethal concentration was calculated.

MLC (µg/mL)	*T. vaginalis* Total N (%)	*T. vaginalis* (Vietnam) N (%)	*T. vaginalis* (Italy) N (%)
≤4.3 µg/mL	48 (75 %)	34 (72%)	14 (82%)
8.6 µg/mL	11 (17%)	9 (19%)	2 (12%)
≥17.1 µg/mL	5 (8%)	4 (9%)	1 (6%)

## Data Availability

The data presented in this study are available in the article and in supplementary material.

## Supplementary Materials

The following supporting information can be downloaded at: https://www.mdpi.com/article/10.3390/antibiotics11060812/s1, Table S1: resistance gene expression in *T. vaginalis* with and without *Mycoplasma* symbionts.
